# Connectivity alterations underlying the breakdown of pseudoneglect: New insights from healthy and pathological aging

**DOI:** 10.3389/fnagi.2022.930877

**Published:** 2022-09-01

**Authors:** Chiara Bagattini, Marco Esposito, Clarissa Ferrari, Veronica Mazza, Debora Brignani

**Affiliations:** ^1^Neurophysiology Lab, IRCCS Istituto Centro San Giovanni di Dio Fatebenefratelli, Brescia, Italy; ^2^Unit of Statistics, IRCCS Istituto Centro San Giovanni di Dio Fatebenefratelli, Brescia, Italy; ^3^Center for Mind/Brain Sciences CIMeC, University of Trento, Rovereto, Italy; ^4^Department of Clinical and Experimental Sciences, University of Brescia, Brescia, Italy

**Keywords:** visuospatial bias, Alzheimer’s disease, mild cognitive impairment, fronto-parietal network, interhemispheric connectivity, multiple-objects enumeration

## Abstract

A right-hemisphere dominance for visuospatial attention has been invoked as the most prominent neural feature of pseudoneglect (i.e., the leftward visuospatial bias exhibited in neurologically healthy individuals) but the neurophysiological underpinnings of such advantage are still controversial. Previous studies investigating visuospatial bias in multiple-objects visual enumeration reported that pseudoneglect is maintained in healthy elderly and amnesic mild cognitive impairment (aMCI), but not in Alzheimer’s disease (AD). In this study, we aimed at investigating the neurophysiological correlates sustaining the rearrangements of the visuospatial bias along the progression from normal to pathological aging. To this aim, we recorded EEG activity during an enumeration task and analyzed intra-hemispheric fronto-parietal and inter-hemispheric effective connectivity adopting indexes from graph theory in patients with mild AD, patients with aMCI, and healthy elderly controls (HC). Results revealed that HC showed the leftward bias and stronger fronto-parietal effective connectivity in the right as compared to the left hemisphere. A breakdown of pseudoneglect in patients with AD was associated with both the loss of the fronto-parietal asymmetry and the reduction of inter-hemispheric parietal interactions. In aMCI, initial alterations of the attentional bias were associated with a reduction of parietal inter-hemispheric communication, but not with modulations of the right fronto-parietal connectivity advantage, which remained intact. These data provide support to the involvement of fronto-parietal and inter-parietal pathways in the leftward spatial bias, extending these notions to the complex neurophysiological alterations characterizing pathological aging.

## Introduction

Neurologically healthy individuals exhibit a leftward visuospatial bias during perceptual judgment tasks, also known as pseudoneglect. The bias consists of better performance for stimuli that appear in the left hemifield (Bowers and Heilman, 1980; Jewell and McCourt, 2000). Pseudoneglect has been typically assessed with the same tasks used to examine neglect in clinical populations, such as line and shape bisection tasks, cancelation, and grayscale tasks (Bowers and Heilman, 1980; Jewell and McCourt, 2000; Sosa et al., 2010; Brooks et al., 2014). Recent studies have shown evidence of leftward visuospatial bias in non-typical paradigms (for visual search tasks see Nicholls et al., 2014, 2017). In this context, we have recently found a left visual field advantage in the performance of a multiple-objects enumeration task in healthy elderly people (Brignani et al., 2018). How aging affects the pseudoneglect phenomenon is still a debated issue with mixed results: whereas several studies reported a reduction of the leftward bias or even a rightward shift of the bias in healthy older adults (Fujii et al., 1995; Failla et al., 2003; Barrett and Craver-Lemley, 2008; Schmitz and Peigneux, 2011; Benwell et al., 2014b), other studies reported no effect of aging on the pseudoneglect or even a stronger leftward bias with increasing age (De Agostini et al., 1999; Varnava and Halligan, 2007; Brooks et al., 2016; Friedrich et al., 2016). Understanding the neural mechanisms of visuospatial bias represents an intriguing hint to extend existing knowledge on brain activity lateralization (Learmonth and Papadatou-Pastou, 2021). In fact, early theoretical models of visual attention have emphasized the right hemispheric dominance to account for normal and pathological visuospatial biases (Heilman and Van Den Abell, 1980; Mesulam, 1981). Evidence of a double fronto-parietal attention-related system, encompassing more right-lateralized ventral and bilateral dorsal activations (Corbetta and Shulman, 2002), paved the way for research into the neural basis of left visual field superiority. For example, Siman-Tov and colleagues (Siman-Tov et al., 2007) found that a set of brain regions belonging to both fronto-parietal systems were vastly recruited during left-sided stimuli presentation and even more so in right posterior parietal areas. A study by Thiebaut de Schotten and colleagues (Thiebaut De Schotten et al., 2011) first demonstrated that the anatomical correlates of such right functional dominance display a tight link with the individual behavioral bias in that larger key white matter fibers (i.e., superior longitudinal fasciculus; SLF) may unbalance the processing speed and thus the final visual field effect. Subsequent studies confirmed the crucial relationship between these structural connectivity measures within the right hemisphere and the extent of pseudoneglect (Wu et al., 2016; Cazzoli and Chechlacz, 2017). Accordingly, damage to these anatomical connections has been proved to yield symptoms of left hemispatial neglect (Corbetta and Shulman, 2011; Bartolomeo et al., 2012; Thiebaut De Schotten et al., 2014), effectively producing a rightward visuospatial bias. Evidence from transcranial magnetic stimulation (TMS) studies in healthy individuals targeting right brain regions typically lesioned in neglect patients showed a temporary reduction of the pseudoneglect phenomenon, with a rightward shift of the bias, together with changes in neural networks underlying the orienting of visuospatial attention (Fierro et al., 2000, 2001; Ricci et al., 2012; Salatino et al., 2014; Bagattini et al., 2015). In addition, the right hemisphere dominance has also been ascribed to the properties of the inter-hemispheric connections between parietal areas, whether they implicate inhibitory or excitatory mechanisms (Bloom and Hynd, 2005). Thus, the functional and structural connectivity advantage of the right hemisphere has been put forward as an empirical explanation of the left visual field superiority (i.e., pseudoneglect) in various studies (Siman-Tov et al., 2007; Chechlacz et al., 2015; Gigliotta et al., 2017). Interestingly, Gigliotta and colleagues (Gigliotta et al., 2017) showed that the fronto-parietal connectivity advantage within the right hemisphere per se was not sufficient to induce pseudoneglect: through the development of simulated neurorobots controlled by artificial neural networks with different constraints, they showed that transcallosal inhibitory connections, together with a greater number of ventral-to-dorsal interactions, were necessary to generate pseudoneglect similar to that measured in human subjects.

The study by Brignani and colleagues (Brignani et al., 2018) also reported clues on pathological aging: while amnesic mild cognitive impairment patients (aMCI) maintained pseudoneglect, patients with Alzheimer’s disease (AD) showed no difference in the performance of the same visual enumeration task between the two visual fields at any numerosity. AD is commonly considered as a memory syndrome, with onset clinical symptoms mainly regarding episodic memory impairment. A growing number of findings revealed that impaired attentional abilities accompany memory deficits among the first clinical manifestations (Finke et al., 2013). While some domains of attention have been explored more than others (Greenwood et al., 1993; Perry and Hodges, 1999; Finke et al., 2013), the alterations of visuospatial biases occurring in AD have often been overlooked and the existing studies provide contrasting results. In addition to early single-case studies on advanced AD with right- and left-side neglect symptoms (Bartolomeo et al., 1998; Venneri et al., 1998), visuospatial difficulties in moderate AD patients have been reported to occur over either visual field, according to which hemisphere mostly exhibited cortical hypometabolism (Meguro et al., 2001; Redel et al., 2012; Sorg et al., 2012). Explanations of AD patients’ spatial behavior should not exclusively be restricted to evidence of metabolic brain activations, which fail to capture the potential connectivity alterations in neural pathways. Neuropathology in AD is in fact characterized by a substantial number of amyloid plaques and neurofibrillary tangles within and across associative areas, as well as by abnormalities in long-range association fibers which have been observed in a number of diffusion tensor imaging (DTI) studies (for a review see Sexton et al., 2011; Pievani et al., 2014), with a progressive decrease in myelination and loss of axons as disease severity advances (Mayo et al., 2017). Currently, the literature on different AD phenotypes has provided no definitive evidence to assume a specific group-level hemispheric asymmetry during the neurodegenerative phases, although the asymmetric pattern may be identified at the individual level (Frings et al., 2015). In addition, decreased interhemispheric connectivity has been consistently observed in patients with AD in the form of marked atrophy and impaired integrity of the corpus callosum (CC; Rose et al., 2000; Wang et al., 2015). These structural imaging studies are in accordance with the proposed “split-brain” model to account for the breakdown of interhemispheric connectivity in AD (Reuter-Lorenz and Mikels, 2005).

On these grounds, the aim of this study was to investigate the neurophysiological correlates subtending the rearrangements of the visuospatial bias in a given sample of patients with a clinical diagnosis of AD dementia, in comparison to aMCI and healthy older adults. By exploring the correspondence between the presence/absence of visuospatial bias and the pattern of intra- and inter-hemispheric connectivity within each group of participants, we expected to acquire valuable insights into the neural underpinnings of pseudoneglect. If these connections contributed to pseudoneglect, we predicted to find right hemispheric dominance in the elderly with pseudoneglect, but not in patients with dementia without pseudoneglect, and altered parietal inter-hemispheric connections in the latter group.

We recorded EEG activity during a multiple-objects enumeration task, already used in our prior report (Brignani et al., 2018), and analyzed effective connectivity in the theta band adopting indexes from graph theory: the connections along the fronto-parietal network and the inter-hemispheric divisibility were used as specific primary measures in contrast to the more general degree index used as a control measure (a detailed description of the graph theory indexes is provided in the methods section). We decided to compute these indexes from theta band frequency because previous studies in both animals and humans assigned a key role to this rhythm in the long-range synchronization of neural activity between anterior and posterior regions of the fronto-parietal network (Sellers et al., 2016; Fiebelkorn et al., 2018). Furthermore, abnormal fronto-parietal theta waves can be a sign of neglect syndrome in right-damaged patients (Yordanova et al., 2017), supporting several findings on the correlation between spatial attention tasks and theta oscillations along this network (Brázdil et al., 2013; Daitch et al., 2013; Dombrowe and Hilgetag, 2014; Park et al., 2019).

## Material and methods

### Participants

Three age-matched groups of participants were included in this study: 14 patients with a clinical diagnosis of mild AD (seven females, mean age = 74.9, SD = 6.0, range = 63–83), 15 amnesic patients with MCI (six females, mean age = 74.5, SD = 5.9, range = 60–81), and 14 healthy elderly controls (HC; 9 females, mean age = 70.9, SD = 3.8, range = 67–82). Data from these participants were included in Bagattini et al. (2017) and partly in Brignani et al. (2018). Clinical diagnosis of AD and amnesic MCI was done by expert neurologists or geriatricians addressing medical history, clinical examination, neuropsychological testing, and imaging results such as computed tomography, MRI, or PET. Patients with AD were diagnosed as suffering from probable AD according to the National Institute of Neurological and Communicative Disorders and Alzheimer’s disease and Related Disorders Association (NINCDS-ADRDA) criteria (McKhann et al., 1984). Patients with AD were identified as eligible for participating in the study if their Mini Mental State Examination (MMSE) score was greater or equal to 20, Clinical Dementia Rating (CDR) scale score was less or equal to 2 and their Hachinski Ischemia score was less or equal to 4. All patients had been on a stable dose of cholinesterase inhibitors (donepezil or rivastigmine) for at least 3 months before participation in the study. Patients with MCI were diagnosed according to the criteria proposed by Petersen (2004). MCI were recruited if their MMSE score was greater or equal to 24, CDR scale score was equal to 0.5, and their Hachinski Ischemia score was less or equal to 4. Patients with potentially confounding medical, neurological, or psychiatric disorders, which could account for the onset of dementia, were not included in the study. Inclusion criteria for HC were the absence of the previous history of neurological or psychiatric problems and an MMSE score between 24 and 30. A comprehensive neuropsychological evaluation was administered to confirm the absence of any cognitive impairment. Exclusion criteria were the presence of medical, neurological, or psychiatric disorders that might interfere with the study. Prior to the beginning of the experiment, all participants gave their written informed consent. All the procedures were approved by the Ethics Committee of the IRCCS San Giovanni di Dio Fatebenefratelli Scientific Institute (Brescia, Italy), and were performed accordingly to the Declaration of Helsinki for research involving human subjects. The demographic and clinical characteristics of the participants are reported in Table 1.

**TABLE 1:** Demographic and age- and education-adjusted neuropsychological characteristics of the three groups of participants (AD, aMCI, and HC) were reported as mean (± SD).

	AD	MCI	HC	*F*	*p*	AD vs. MCI	MCI vs. HC	AD vs. HC
Age (years)	74.79 (6.25)	74.47 (6.05)	70.85 (3.90)	2.213	0.12	–	–	–
Education (years)	7.21 (2.67)	8.40 (3.07)	9.21 (3.68)	1.416	0.26	–	–	–
CDR	0.93 (0.51)	0.47 (0.13)	0.00 (0.00)					
MMSE	21.79 (1.35)	26.21 (1.84)	27.76 (2.12)	41.596	***	***	0.08	***
GDS	5.50 (4.67)	6.00 (3.21)	4.57 (3.82)	0.473	0.63	–	–	–
***Neuropsychological profile***								
RAVLT – immediate recall	28.65 (6.61)	37.57 (9.05)	46.48 (5.08)	21.711	***	**	**	***
RAVLT – delayed recall	2.61 (1.89)	7.21 (3.22)	10.36 (2.09)	34.235	***	***	**	***
Episodic memory	2.04 (1.22)	7.83 (4.92)	14.46 (3.44)	42.321	***	***	***	***
ROCF – copy	29.44 (8.29)	33.48 (5.06)	35.86 (1.55)	4.471	*	0.18	0.58	*
ROCF – recall	7.23 (3.99)	12.87 (7.72)	18.30 (4.65)	34.235	***	*	*	***
Digit span forward	5.59 (0.96)	5.63 (0.90)	5.68 (0.99)	0.031	0.97	–	–	–
Spatial span	5.59 (0.96)	4.32 (0.93)	5.07 (0.77)	4.207	*	0.93	*	0.10
Attentive matrices	39.04 (14.84)	45.37 (7.75)	48.66 (6.16)	3.146	*	0.29	0.77	*
TMT A	59.36 (34.24)	47.40 (32.67)	22.71 (7.95)	6.319	**	0.586	0.06	**
Stroop test – errors	1.57 (2.46)	2.13 (3.13)	0.29 (0.95)	2.211	0.12	–	–	–
Stroop test – time	40.90 (30.34)	24.61 (19.42)	15.89 (7.28)	4.840	**	0.15	0.61	*
RCPM47	28.61 (6.61)	30.70 (3.73)	32.79 (3.17)	3.354	*	0.48	0.48	*
Phonemic verbal fluency	28.79 (4.74)	28.80 (7.86)	40.93 (9.68)	11.716	***	1.00	***	***

Results of the ANOVA model (F and p-values) and post-hoc comparisons with Sidak correction (p-values) between the groups are reported. Asterisks indicate significant differences (*p ≤ 0.05; **p ≤ 0.01; ***p ≤ 0.001). CDR, Clinical Dementia Rating; MMSE, Mini Mental State Examination; GDS, Geriatric Depressive Scale; RAVLT, Rey Auditory Verbal Learning Test; ROCF, Rey-Osterrieth Complex Figure; TMT, Trail Making Test; RCPM47, Raven Colored Progressive Matrices.

### Stimuli and procedure

**FIGURE 1:**
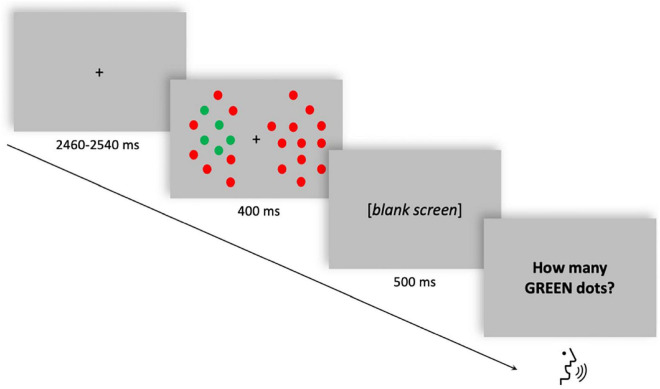
Single trial structure of the multiple-objects enumeration task. Example of a trial with five targets (green dots) appearing on the left visual field among distractors (red dots). Participants had to verbally report the number of targets (from 1 to 6).

Participants were required to perform an enumeration task in which they had to verbally report a variable number of targets (from 1 to 6 green dots) among distractors (red dots) presented either to the left or to the right of a fixation dot. Visual enumeration is characterized by the so-called “subitizing phenomenon” (Kaufman and Lord, 1949), which is the fast and accurate performance when reporting up to approximately three items, compared to enumerating larger quantities (“counting”). The total number of dots was held constant while the number of targets varied randomly across trials. Each trial began with a random interval (ranging from 2,460 to 2,540 ms) displaying the fixation dot. The stimulus array was then displayed for 400 ms. After a blank frame lasting 500 ms, the response screen was displayed until the participant’s verbal response (Figure 1). The verbal response of the participant was recorded by the experimenter using a response box. A total of 600 trials (10 blocks, 60 trials each) were delivered. The stimuli were generated, and responses were recorded using the E-Prime 2 software (Psychology Software Tools, Pittsburgh, PA, United States). For a comprehensive description of stimuli and procedure, see Pagano et al. (2015).

### Electrophysiological recordings

Electroencephalogram (EEG) was continuously recorded from an ActiCAP cap with 27 active Ag/AgCl electrodes (Brain Products, GmbH, Munich, Germany) placed according to the 10–20 International System and comprising: Fp1, Fp2, F7, F3, Fz, F4, F8, FCz, T7, C3, Cz, C4, T8, CP5, CP6, P7, P3, Pz, P4, P8, PO7, PO9, PO8, PO10, O1, Oz, and O2. The signal was referenced online to the right mastoid (RM) and the ground electrode was placed over AFz. Horizontal and vertical eye movements were detected with electrodes placed, respectively, at the left and right canthi and above and below the right eye. The EEG was recorded at a 1,000 Hz sampling rate with a time constant of 10 s as a low cut-off filter and a high cut-off of 250 Hz.

### Data processing

#### Pre-processing

EEG traces were band-pass filtered in the 1–45 Hz range and downsampled to 256 Hz. Data were then segmented according to the stimuli onset in the time window (from 0 to 400 ms) usually considered in the investigation of visuospatial bias (Benwell et al., 2014a; Learmonth et al., 2017) and involved in the attentive enumeration processes (Mazza and Caramazza, 2011; Pagano and Mazza, 2012; Pagano et al., 2014). Derived segments were classified in four experimental conditions as a function of target numerosity (low numerosity: from 1 to 3 targets; high numerosity: from 4 to 6 targets) and visual hemifield (left, right). Target numerosity was classified as a two-level factor (low and high) as in a previous study the factor visual hemifield showed a significant interaction with numerosity, suggesting that the pseudoneglect effect was modulated by the number of targets presented, with a greater leftward bias for high numerosity (Brignani et al., 2018). Furthermore, the mean subitizing span (i.e., the number of targets that can be processed simultaneously before counting processes come into play) for patients with AD, patients with aMCI, and HC was 3.17 targets (Bagattini et al., 2017). A semi-automatic procedure based on the threshold criterion (± 100 μV) was then applied to remove muscular and ocular artifacts. Only trials free of artifacts and associated with correct answers were subjected to connectivity analysis. To level out the number of trials included in the analysis across the three experimental groups, we randomly selected 60 correct trials among the total for each experimental condition and each subject.

#### Connectivity estimation

Effective connectivity was estimated in the selected trials by means of Partial Directed Coherence (PDC; Baccalá and Sameshima, 2001), a spectral multivariate estimator, relying on the concept of Granger causality (Granger, 1969), which provides strength and direction of the causal links between different brain areas. Estimated connectivity patterns were first averaged in the theta (3–7 Hz) frequency band and then statistically validated through an asymptotic statistic approach (alpha = 0.05) against the null case (Toppi et al., 2016). Statistical thresholds were then used to convert connectivity matrices into binary adjacency matrices on which extracting several graph indices describing local and global network properties. In particular:


(1) $$G_{i\,j}\,\left(f,t\right)=\left\{ \begin{array}{c} 1\to A_{i\,j}\,\left(f,t\right)\geq \tau_{i\,j}\,\left(f,t\right)\\ 0\to A_{i\,j}\left(f,t\right)<\tau_{i\,j}\left(f,t\right) \end{array}\right.$$

where *G*_*ij*_ and *A*_*ij*_ represent the entry *(i,j)* of the adjacency matrix *G* and the PDC matrix *A*, respectively, and τ_*ij*_ is the corresponding statistical threshold. The use of a statistical threshold in the adjacency matrix extraction process allows for the extraction of reliable graph theory indices and prevents the detection of false network properties (Toppi et al., 2012).

Before moving to the graph theory analysis, connectivity patterns were scaled from the level of electrodes to the macro-areas one (i.e., ROIs). In particular, the contributions of neighboring electrodes were fused through an averaging process including the following ROIs: right-frontal (FP2, F4, and F8), left-frontal (FP1, F3, and F7), frontal (Fz and FCz), left-central (C4), right-central (C3), central (Cz), right-parietal (CP6, P8, P4, and PO8), left-parietal (CP5, P7, P3, and PO7), and occipital (O1, O2, and Oz). Graph indices were thus derived on the scaled corresponding adjacency matrices. The scaling process allowed to reduce the spurious effects in networks involving neighboring electrodes due to the volume conduction effect. Indices extracted from the macro-areas were normalized to take into account the different number of electrodes in each macro-area.

#### Graph theory indices

To test our hypothesis, we first considered the local indices “fronto-parietal connection” and “divisibility,” which describe the communication between intra- and inter-hemispheric certain scalp areas, respectively. To confirm the specificity of the results, we also considered the local index “degree,” which discloses a more widespread involvement of a specific node of the network.

“Fronto-parietal connections” is an index quantifying the number of connections exchanged between frontal and parietal scalp areas normalized over the total number of connections possibly connecting those two areas. It can be defined as:


(2) $$F\,P\,C=\frac{\sum_{i\,\epsilon \,G_{A}}\sum_{j\,\epsilon \,G_{P}}g_{i\,j}+\sum_{i\,\epsilon \,G_{P}}\sum_{j\,\epsilon \,G_{A}}g_{i\,j}}{N_{A}^{2}+N_{P}^{2}}$$

where *G_A_* represents the set of nodes belonging to the anterior areas (*n* = *N*_*A*_) and *G_P_* represents the set of nodes belonging to the posterior areas (*n* = *N*_*P*_). The index was computed separately for left and right hemispheres.

“Divisibility” quantifies how well the general connectivity network can be divided into two sets of nodes, corresponding to two different brain areas. It can be computed as follows (Newman, 2006):


(3) $$D=\frac{W}{\sum_{i,j\,\epsilon \,N}w_{i\,j}\,\left[1-\delta \,\left(C_{i},C_{j}\right)\right]+k}$$

where *C_i_* indicates the community to which the node *i* belongs, the δ function yields 1 if vertices *i* and *j* are in the same community (i.e., in the same area) and 0 otherwise; *W* is the total weight of the network, that is the sum of all arc weights in the graph; *k* is a positive constant (here set equal to *W*) to avoid possible divergence of *D*. Here divisibility index was computed between left and right hemispheres, separately for frontal and parietal areas.

The “degree” of a node is the number of links connected directly to it. In directed networks, the indegree is the number of inward links and the outdegree is the number of outward links (Sporns et al., 2004). It can be defined as follows:


(4) $$k_{f}=\sum_{j\in N,j\neq f}g_{f\,j}+\sum_{i\in N,i\neq f}g_{i\,f}$$

where *g*ij** represents the entry *ij* of the Adjacency matrix *G.* The degree of a specific electrode was normalized by the network density to capture local changes and not a general increase/decrease of the network density. The degree index was computed for each macro-area in the network. All the indices were extracted for each experimental condition and each subject and then fed into the statistical analysis described below.

### Statistical analyses

To test the hypothesis of a right hemisphere connectivity advantage in participants exhibiting pseudoneglect, statistical analyses on behavioral and connectivity data (fronto-parietal connections and degree) were conducted separately for each group of subjects (HC, aMCI, and AD). The visuospatial bias was investigated by subtending the arcsine-transformed *accuracy rates* (Snedecor and Cochran, 1967) to repeated measures ANOVAs with *visual field* (2 levels: left vs. right) and *numerosity* (2 levels: low vs. high) as within-subject factors. *Fronto-parietal connections* were entered into repeated measures ANOVAs with *visual field* (two levels: left vs. right), *numerosity* (two levels: low vs. high), and *hemisphere* (two levels: left vs. right) as within-subjects factors. Repeated measures ANOVAs on *degree index* included the within-subjects factors *visual field* (2 levels: left vs. right), *numerosity* (2 levels: low vs. high), *hemisphere* (2 levels: left vs. right), and *ROI* (2 levels: frontal vs. parietal).

To investigate whether patients with AD displayed alterations in inter-hemispheric parietal connectivity in comparison to healthy elderly, and thus demonstrating a crucial role of inter-hemispheric communication to generate pseudoneglect, the *divisibility index* was entered into two mixed-model ANOVAs separately for the frontal and parietal areas, with *group* (three levels: AD vs. aMCI vs. HC) as between-subject factor, *visual field* (two levels: left vs. right), and *numerosity* (two levels: low vs. high) as within-subject factors. The Greenhouse-Geisser epsilon correction factor was applied, when appropriate, to compensate for possible effects of non-sphericity in the measurements. Post-hoc comparisons were performed with Sidak correction for multiple comparisons.

## Results

### Healthy controls

#### Behavioral data

**FIGURE 2:**
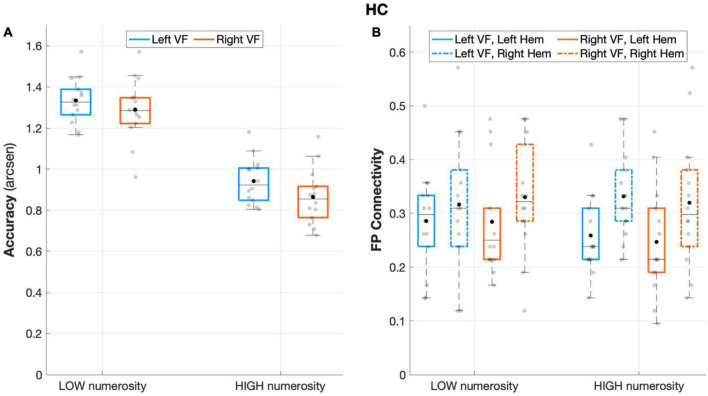
Behavioral and connectivity results in the group of healthy controls. **(A)** Mean accuracy values (arcsen) of healthy controls in the multiple object enumeration task plotted as a function of target numerosity for left (light blue) and right (orange) visual field presentation. Mean values (black dots) are displayed over the 95% confidence interval (light-blue and orange bars) and standard deviation (vertical dashed lines). **(B)** Fronto-parietal connections in healthy controls plotted as a function of visual field (light blue for left VF, orange for right VF) and target numerosity (low, high) for left (solid lines) and right (dashed orange) hemisphere. Mean values (black dots) are displayed over the 95% confidence interval (light-blue and orange bars) and standard deviation (vertical dashed lines).

The repeated measures ANOVA on accuracy rates revealed a significant main effect of *numerosity* (*F*(1,13) = 146.708, *p* < 0.001, *partial eta squared* = 0.919), and *visual field* (*F*(1,13) = 7.037, *p* = 0.02, *partial eta squared* = 0.351), with a non-significant interaction effect (*visual field* X *numerosity*: *F*(1,13) = 0.619, *p* = 0.445, *partial eta squared* = 0.045), thus showing an overall increased accuracy rates for low numerosity and for left-sided target presentation (Figure 2A).

#### Intra-hemispheric connectivity data

The analysis of fronto-parietal connections in healthy controls revealed a main effect of the *hemisphere* that neared statistical significance (*F*(1,13) = 4.403 *p* = 0.056, *partial eta squared* = 0.253), with greater fronto-parietal connections in the right relative to the left hemisphere (Figure 2B). No other main or interaction effect was found between the other factors (all *p* > 0.27).

When considering the *degree* as dependent variable such asymmetrical connectivity was not apparent (*hemisphere*: *F*(1,13) = 0.005, *p* = 0.94, *partial eta squared* = 0.00). For this analysis, none of the other main effects, such as *ROI*, *visual field*, and *numerosity*, was substantial enough to yield any influence on the degree associated with either hemisphere (all *p* > 0.16).

### Amnesic mild cognitive impairment patients

#### Behavioral data

**FIGURE 3:**
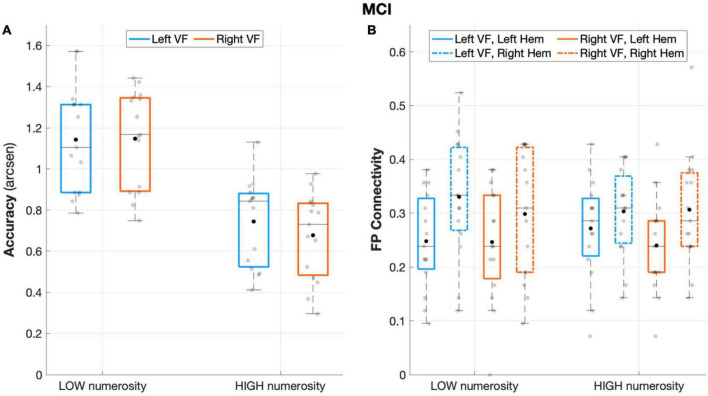
Behavioral and connectivity results in the group of patients with MCI. **(A)** Mean accuracy values (arcsen) of MCI in the multiple object enumeration task plotted as a function of target numerosity for left (light blue) and right (orange) visual field presentation. Mean values (black dots) are displayed over the 95% confidence interval (light-blue and orange bars) and standard deviation (vertical dashed lines). **(B)** Fronto-parietal connections in MCI plotted as a function of visual field (light blue for left-VF, orange for right-VF) and target numerosity (low, high) for the left (solid line) and right (dashed line) hemispheres. Mean values (black dots) are displayed over the 95% confidence interval (light-blue and orange bars) and standard deviation (vertical dashed lines).

The repeated measures ANOVA on accuracy rates revealed a significant main effect of *numerosity* (*F*(1,14) = 170.484, *p* < 0.001, *partial eta squared* = 0.924), with a better performance during low numerosity trials. Although the overall performance was not affected by the *visual field* factor (*F*(1,14) = 1.902, *p* = 0.190, *partial eta squared* = 0.120), the interaction effect *visual field* X *numerosity* (*F*(1,14) = 7.573, *p* = 0.016, *partial eta squared* = 0.350) and subsequent post-hoc showed that aMCI patients performed significantly better when targets appeared in the left visual field with high (*p* = 0.017) but not low (*p* = 0.87) numerosity (Figure 3A).

#### Intra-hemispheric connectivity data

The aMCI group showed a significant main effect of *hemisphere* (*F*(1,14) = 8.487, *p* = 0.011, *partial eta squared* = 0.377), with greater fronto-parietal connections in the right relative to the left hemisphere (Figure 3B). No other significant effect emerged from the analyses on fronto-parietal connections (all *p* > 0.29).

The analyses on the *degree* index revealed a significant main effect of *ROI* (*F*(1,14) = 7.155, *p* = 0.018, *partial eta squared* = 0.338) indicating major overall recruitment of frontal areas. No other significant effects emerged (all *p* > 0.23) except for the *hemisphere* × *visual field* interaction effect (*F*(1,14) = 7.87, *p* = 0.014, *partial eta squared* = 0.360), which indicated a greater degree of connectivity in the left hemisphere during left-sided target presentation, although not surviving to the Sidak correction (*p* > 0.05).

### Alzheimer’s disease patients

#### Behavioral data

**FIGURE 4:**
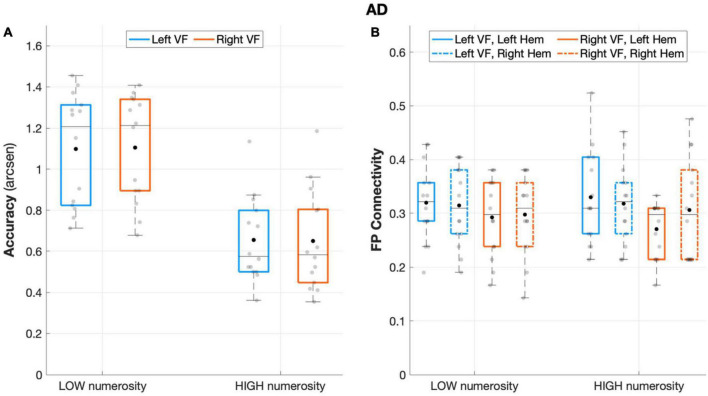
Behavioral and connectivity results in the group of patients with AD. **(A)** Mean accuracy values (arcsen) of AD in the multiple object enumeration task plotted as a function of target numerosity for left (light blue) and right (orange) visual field presentation. Mean values (black dots) are displayed over the 95% confidence interval (light-blue and orange bars) and standard deviation (vertical dashed lines). **(B)** Fronto-parietal connections in AD are plotted as a function of visual field (light blue for left-VF, orange for right-VF) and target numerosity (low, high) for the left (solid line) and right (dashed line) hemispheres. Mean values (black dots) are displayed over the 95% confidence interval (light blue and orange bars) and standard deviation (vertical dashed lines).

The analysis on AD sample revealed no main or interaction effect of visual field (*visual field*: *F*(1,13) = 0.002, *p* = 0.963, *partial eta squared* = 0.00; *visual field* × *numerosity*: *F*(1,13) = 0.204, *p* = 0.659, *partial eta squared* = 0.015), that is, neither the side of targets presentation nor its interaction with numerosity affected accuracy rates. Nevertheless, AD performance remained sensitive to the number of targets (*numerosity*: *F*(1,13) = 89.869, *p* < 0.001, *partial eta squared* = 0.874), with overall greater accuracy in low numerosity trials (Figure 4A).

#### Intra-hemispheric connectivity data

Patients with AD failed to show fronto-parietal connections asymmetry, as the factor *hemisphere* did not show a significant effect (*F*(1,13) = 0.124, *p* = 0.730, *partial eta squared* = 0.009), along with the rest of the other factors (all *p* > 0.11; Figure 4B).

Unlike the HC, but similar to the aMCI group, the analyses on the *degree* index revealed a significant main effect of *ROI* (*F*(1,13) = 14.43, *p* = 0.002, *partial eta squared* = 0.526), indicating major overall recruitment of frontal areas. No other significant effects emerged (all *p* > 0.1), except for the *ROI* × *visual field* × *numerosity* interaction effect (*F*(1,13) = 5.33, *p* = 0.038, *partial eta squared* = 0.291), suggesting a greater involvement of frontal areas specifically when a higher number of targets appeared on the right visual field, although this effect did not survive to the Sidak correction (*p* = 0.086).

### Inter-hemispheric connectivity

**FIGURE 5:**
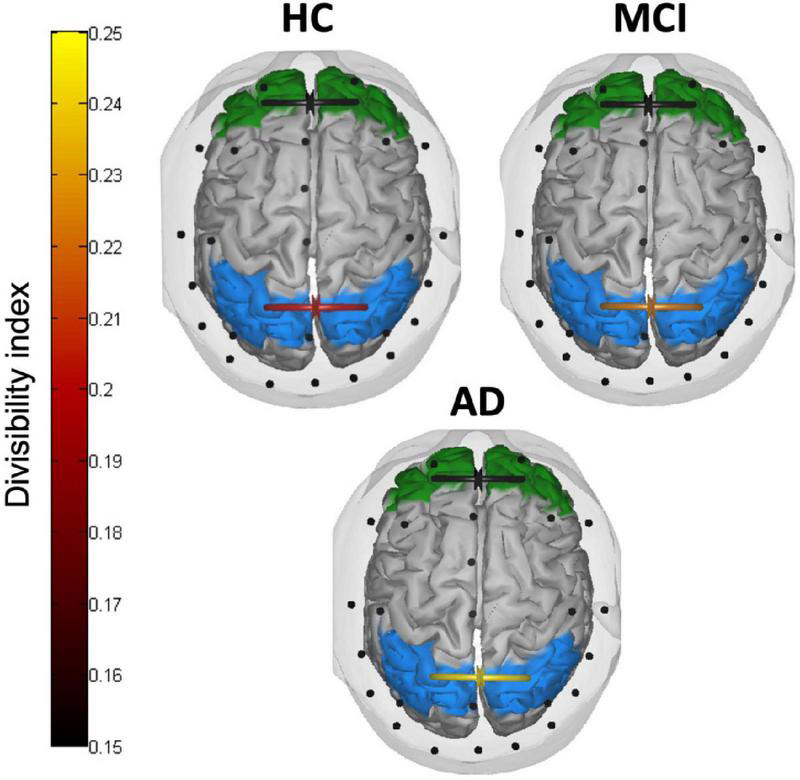
Inter-hemispheric connectivity results. Divisibility index in frontal (green) and parietal (light blue) areas in HC, patients with MCI, and patients with AD. Higher (lighter) values indicate higher divisibility between the two sets of nodes.

Figure 5 depicts the divisibility index in the three groups of participants. The two mixed ANOVAs on the divisibility index revealed no significant effects between the three groups in frontal areas (all *p* > 0.093), while in parietal areas the main effect of the group reached a significance level (*F*(2,40) = 3.302, *p* = 0.047, *partial eta squared* = 0.142). The divisibility index followed an uptrend (higher values indicating higher divisibility) from normal aging, through aMCI, to AD, with post-hoc comparisons showing a significant difference between HC and patients with AD (*p* = 0.041). Neither a difference between low and high *numerosity* condition (*F*(1,40) = 0.015, *p* = 0.902, *partial eta squared* = 0.00) nor interactions between *group* and *numerosity* (*F*(2,40) = 1.712, *p* = 0.193, *partial eta squared* = 0.079) emerged.

## Discussion

To extend existing knowledge on the neural mechanisms underlying the phenomenon of pseudoneglect, this study provides interesting hints to clarify what connectivity alterations are associated with its breakdown in pathological aging. Pseudoneglect is a behavioral phenomenon that arises naturally in healthy people and comprises a set of left-sided spatial biases (Bowers and Heilman, 1980; Jewell and McCourt, 2000; Charles et al., 2007). In this study, we aimed at investigating the neurophysiological correlates sustaining the modifications of the visuospatial bias in a visual enumeration task along the progression from normal to pathological aging. We explored the correspondence between the presence/absence of pseudoneglect and the pattern of intra- (fronto-parietal) and inter- (parieto-parietal and fronto-frontal) hemispheric connectivity dynamics within groups of healthy elderly, patients with aMCI, and patients with mild AD.

The behavioral results showed that attentive enumeration of multiple objects is subjected to leftward visuospatial bias in normal aging, whereas no pseudoneglect-like behavior emerged in the sample of patients with mild AD, as previously reported (Brignani et al., 2018). The results do not allow us to rule out that the maintenance of the leftward visuospatial bias in healthy aging was specific to the present enumeration task, and not generalizable to other tasks typically adopted in the study of pseudoneglect (e.g., line bisection). For instance, a right hemisphere specialization has been suggested for the subitizing component of enumeration (Warrington and James, 1967; Kimura and Durnford, 1974; Pasini and Tessari, 2001; Jackson and Coney, 2004; but see Butterworth, 1999). It has been shown, however, that the left bias found in enumeration tasks concerns the visuospatial processing required for the task, rather than enumeration abilities per se (Kimura, 1966; Boles, 1986). Altogether, these findings suggest that the nature of the lateralization in the present task may be the same as the one found in typical pseudoneglect paradigms such as line bisection.

In line with prior neuroimaging studies on healthy adults (Thiebaut De Schotten et al., 2011; Wu et al., 2016; Cazzoli and Chechlacz, 2017), the analysis of theta band effective connectivity revealed that fronto-parietal pathways within the right hemisphere were overall stronger in participants showing a leftward bias (i.e., HC participants). On the contrary, patients with AD who did not display a behavioral left visual field advantage failed to show such fronto-parietal connections asymmetry. A right-hemisphere dominance in visuospatial attention has been invoked as the most prominent neural feature of pseudoneglect, especially as compared to activations during non-spatial tasks (Fink et al., 2000; Çiçek et al., 2009; Cavézian et al., 2012; Cai et al., 2013; Zago et al., 2017; Chen et al., 2020). However, the neurophysiological meaning of such right-hemisphere dominance is still controversial. The present results are well aligned with the general evidence that AD degeneration affects large-scale networks and particularly those connecting anterior and posterior brain regions (Delbeuck et al., 2003). A reduction of fronto-parietal connectivity in patients with AD has been reported in several electrophysiological and imaging studies (Horwitz et al., 1987; Berendse et al., 2000; Neufang et al., 2011; Zhang et al., 2015). The lateralization of the development of AD pathophysiology has also been investigated by numerous studies, but with discordant results. Several indications support the fact that AD neurodegeneration may affect hemispheres in an asymmetric fashion (Haxby and Rapoport, 1985; Braak et al., 1993; Derflinger et al., 2011; Finke et al., 2013). A recent longitudinal FDG-PET study reported a diverging pattern of lateralized decline in patients with MCI vs. patients with mild AD as compared to healthy elderly: whereas the former showed signs of stronger left hemisphere degeneration, the latter was associated with a right hemisphere prevalence of the glucose hypo-metabolism (Weise et al., 2018). Interestingly, the authors concluded that hemispheric metabolic asymmetries may effectively diminish along the disease progression. Therefore, a stronger right-sided decline of the core cerebral regions of AD pathology (such as the fronto-parietal network) in the mild dementia phases may explain the loss of the right hemisphere connectivity advantage typical of healthy controls (Thiebaut De Schotten et al., 2011), hence the ensuing breakdown of the visuospatial bias. Alternatively, other studies have proposed increased connectivity between regions of the left hemisphere as the possible neural mechanism underlying the loss of right hemisphere dominance in patients with AD (see e.g., Seeley et al., 2007; Agosta et al., 2012).

Based on the comparison between the results obtained from healthy elderly and patients with AD, the right fronto-parietal connectivity advantage might appear as the neural underpinning crucially sustaining the leftward bias. However, findings from the sample of aMCI, which might represent the intermediate condition between HC and patients with AD, provide valuable hints for understanding the neural substrates of the initial spatial bias modifications. In patients with aMCI, indeed, pseudoneglect was present in the high numerosity condition, but not in the low numerosity condition. Consistently, our previous study clearly showed that the magnitude of the leftward bias was modulated by the number of targets to be enumerated, with larger numerosity eliciting a greater advantage for the left visual field (please refer to Figure 2 of Brignani et al., 2018). However, at the electrophysiological level, patients with aMCI showed a similar right fronto-parietal connectivity advantage in both high and low numerosity conditions, irrespective of the difference in the behavioral bias. This finding calls into question the contribution of intra-hemispheric asymmetry in the genesis of pseudoneglect, while a more consistent correspondence may be provided by modulations of inter-parietal transcallosal connections.

The integrity of callosal pathways is essential for the processing and integration of visuospatial information (Schulte and Müller-Oehring, 2010; Berlucchi, 2014) and a crucial role of inter-parietal connections has been asserted to account for the neglect syndrome (e.g., inter-hemispheric rivalry model, Kinsbourne, 1977, 1978, 1987, 1993). Although it is still debated whether the corpus callosum plays an inhibitory or excitatory influence on the contralateral hemisphere during the interhemispheric transfer of information (Bloom and Hynd, 2005), it is reasonable to assume that, whether these connections sustain the right hemisphere dominance in visuospatial processing, a leftward bias begins to be less prominent once these connections fail to properly function. This was indeed the case with the present sample of patients with AD, who showed, consistently with their lack of pseudoneglect, much higher divisibility values as compared to HC. Divisibility quantifies how well a connectivity network can be divided into two sets of nodes (i.e., brain areas) with greater values revealing higher independence between the two. Although this measure cannot specify the nature of the connection itself (i.e., excitatory vs. inhibitory), it allowed us to identify a stark difference between patients with AD and healthy elderly participants in terms of reduced communication between homologous parietal, but not frontal, areas. In the original proposal of AD as a “disconnection syndrome” the cortico-cortical connectivity breakdown involved not only long-range intra-hemispheric connections but also inter-hemispheric pathways (Delbeuck et al., 2003). Indeed, numerous imaging evidence highlighted marked changes in the CC along the continuum of AD pathology with reduced functional and structural connectivity as consistent findings (Reuter-Lorenz and Mikels, 2005; Wang et al., 2006, 2015; Xie et al., 2006; Di Paola et al., 2010; Preti et al., 2012).

The divisibility index observed in the sample of aMCI was amid the extreme values measured in HC and patients with AD, although it did not statistically differentiate from either of the two. Nonetheless, this trend is likely the first physiological sign appearing when leftward spatial bias begins to falter in the early neurodegenerative condition. We speculate that while healthy participants have a functionally preserved inferior parietal lobule where the visual information is quickly integrated across hemispheres to form a leftward-biased topography of the input (Le et al., 2015; Chen et al., 2019), the initial degeneration of transcallosal connections in patients with aMCI may have contributed to affect this early parietal advantage when enumerating small quantities. Conversely, when enumerating larger quantities, some aspects of the evidence accumulation process may have preserved pseudoneglect in patients with aMCI. As the process of evidence accumulation also depends on the strength of externally presented sensory evidence, patients with aMCI may need a stronger visual stimulation (i.e., the area covered by the target stimuli) to be sampled and accumulated as evidence (as compared to healthy elderly) before the threshold to initiate the response is reached (Liu and Pleskac, 2011). In line with this explanation, patients with AD did not present a pseudoneglect-like behavior in enumerating small or large numerosities, which strikingly matches their reduced fronto-parietal asymmetry and inter-parietal connections altogether. As the behavioral attentional bias partially faltered, a reduction of parietal inter-hemispheric communication was observed. Together, the fronto-parietal connectivity advantage of the right over the left hemisphere remained intact. Conversely, the breakdown of the leftward bias was associated with the loss of the fronto-parietal asymmetry and a further inter-hemispheric disconnection.

Although the presence of attentional impairments in AD has been documented using a variety of tasks, the existence of impaired lateralization of visuospatial attentional has remained controversial (Mendez et al., 1997; Bartolomeo et al., 1998; Venneri et al., 1998; Foster et al., 1999; Ishiai et al., 1996, 2000; Redel et al., 2012). Patients with AD displayed a more general inability to distribute attention across both hemifields, with a preference for the right or the left on an individual basis (Redel et al., 2012). We believe that different patterns of perceptual bias observed across studies may depend on the fact that asymmetrical fronto-parietal degeneration in AD is rather frequent at the individual level, explaining rightward or leftward biases when being more pronounced in the right or left hemisphere, respectively.

Finally, connectivity in terms of the degree index served as a control analysis for the specificity of fronto-parietal connections in explaining the visuospatial bias. As expected, no lateralized effects emerged for this connectivity measure. To accurately perform the task (analysis has been performed considering accurate trials only) both patients with aMCI and AD engaged greater bilateral connectivity in frontal areas, as shown in the degree index results. This effect resonates with the evidence of compensatory mechanisms similar to those described by the “compensation-related utilization of neural circuits hypothesis” (CRUNCH; Reuter-Lorenz and Cappell, 2008). This influential model predicts that the over-recruitment of brain areas serves as compensatory activity against deterioration processes (Reuter-Lorenz et al., 2000; Cabeza et al., 2002). Compensatory mechanisms, however, may also be lateralized in the brain as is the case of the contralateral delay activity (CDA), an EEG neural marker of the multiple object enumeration process that was found to be able to capture hemispheric lateralization in older adults (Bagattini et al., 2017; Sander et al., 2019).

Although the present results appear promising, some issues need to be addressed. Longitudinal studies assessing the trajectory of pseudoneglect from youth to old age (and eventually to pathological aging) are needed to verify whether and how visuospatial bias changes throughout the lifespan. Indeed, results from the present cross-sectional study cannot bear out that patients with AD displayed a leftward bias before disease onset. Regarding the research samples, diagnosis of AD was based on clinical criteria only and was not supported by any fluid or imaging biomarkers; thus, also patients whose cognitive deficits could have other causes than AD pathology might have been included. Accordingly, the results might not be specifically due to the presence of AD pathology. At the same time, it is possible that a certain amount of HC participants might fall into the preclinical AD category due to the presence of increased amyloid levels. In this study, HC participants should be considered as “cognitively healthy” individuals. Furthermore, we studied pseudoneglect mainly as a group-level phenomenon whose variations could have gone undetected at an individual level, thus making the generalizability of the results more difficult. Finally, results must be interpreted with some caution as they derive from correlational and not causal measures. Indeed, the association between fronto-parietal and inter-parietal connectivity and the visuospatial bias does not entail a direct cause-and-effect relationship between these variables. However, the fact that the breakdown of pseudoneglect in AD is associated with the absence of a fronto-parietal connectivity advantage and reduced inter-parietal communication does support our hypothesis.

## Conclusion

The present results suggest that the leftward visuospatial bias observed during the execution of a multiple-object enumeration task in older adults was associated with a right fronto-parietal connectivity advantage together with a greater inter-hemispheric parietal communication. Consistently, the breakdown of pseudoneglect in patients with mild AD was sustained by the loss of the fronto-parietal asymmetry along with a reduction of inter-hemispheric parietal interactions. An early alteration of the attentional bias was observed in a sample of patients with aMCI in association with a reduction of parietal inter-hemispheric communication but not with alterations of the right fronto-parietal connectivity advantage that remained intact.

Future studies will need to replicate these findings with more standard tasks for pseudoneglect, such as the line bisection or the landmark task. Replicating this study with different visuospatial tasks will probe the generalizability of the results to the tasks typically adopted in the investigation of pseudoneglect, and thus confirm the multiple-objects visual enumeration paradigm as a valid model to explore the pseudoneglect dynamics. A better understanding of the behavioral variability and the underlying neural mechanisms will aid not only to extend our knowledge of the pseudoneglect phenomenon through healthy and pathological aging but can also have clinical relevance. Shedding new light into compensatory mechanisms and large-scale functional reorganization may pave the way for promoting better cognitive strategies to delay AD attention-related symptoms.

## Data availability statement

The raw data supporting the conclusions of this article will be made available by the authors, without undue reservation.

## Ethics statement

The studies involving human participants were reviewed and approved by Comitato Etico IRCCS San Giovanni di Dio Fatebenefratelli Via Pilastroni, Brescia, Italy. The patients/participants provided their written informed consent to participate in this study.

## Author contributions

CB collected data, analyzed data, and co-wrote the manuscript. ME and CF analyzed data and co-wrote the manuscript. VM and DB conceived the experiment, supervised the entire work, and co-wrote the manuscript. All authors contributed to the article and approved the submitted version.
